# Association between systemic immune-inflammation index and all-cause and CVD mortality in non-elderly diabetic adults

**DOI:** 10.1016/j.clinsp.2025.100739

**Published:** 2025-08-06

**Authors:** Bing Hu, Tong Liu, Yanxiang Sun, Jie Sun, Li Feng, Fei Li

**Affiliations:** Department of Cardiology, Zhongshan City People’s Hospital, Zhongshan, Guangdong, China

**Keywords:** Systemic immune-inflammation index, Diabetes, Cardiovascular disease, Mortality

## Abstract

**Background:**

The Systemic Immune-Inflammation Index (SII) is widely studied for its role in evaluating Cardiovascular Disease (CVD) and mortality. However, its association with all-cause and CVD mortality in non-elderly diabetic adults remains unclear. This study aims to explore the relationship between SII and both all-cause and CVD mortality in non-elderly diabetic adults in the United States.

**Methods:**

Cox proportional hazards models and subgroup analyses were used to assess the link between SII and mortality. Restricted Cubic Splines (RCS) examined the nonlinear association between SII and mortality.

**Results:**

Data from 4680 participants in NHANES (2001‒2018) were analyzed. Over an average follow-up of 98.3 months, there were 625 all-cause deaths and 162 CVD-related deaths. Participants were categorized into two SII groups (higher: > 947.625, lower: ≤ 947.625). Multivariable-adjusted models showed that higher SII levels were significantly associated with an increased risk of both CVD mortality (HR = 3.05; 95 % CI 1.85, 5.01) and all-cause mortality (HR = 1.97; 95 % CI 1.50, 2.58). Subgroup analyses confirmed the consistency of these associations. RCS analysis revealed a nonlinear relationship between SII and all-cause mortality, while the association with CVD mortality was linear.

**Conclusion:**

Higher SII levels in non-elderly diabetic adults in the U.S. are linked to an increased risk of both CVD and all-cause mortality.

## Highlights

· Elevated SII predicts increased CVD/all-cause mortality in US non-elderly adults with diabetes.
· SII > 947.6 elevates CVD mortality risk 3.05-fold (95 % CI 1.85‒5.01).
· Nonlinear SII-all-cause vs. linear SII-CVD mortality links in NHANES cohort.
· Consistent SII-mortality associations across multi-subgroup analyses confirmed.

## Introduction

Diabetes, characterized by hyperglycemia, stands as the most prevalent serious chronic disease today. Type 2 Diabetes Mellitus (T2DM) has emerged as a major health challenge of the 21st century, exerting significant social impacts worldwide. Since the 1980s, T2DM prevalence has surged across almost all regions. The Global Burden of Disease, Injury, and Risk Factor Study (GBD) 2021 estimates that 529 million individuals globally are affected by diabetes, with an age-standardized prevalence of 6.1 %. By 2050, this number is projected to exceed 1.31 billion.^1^

Traditionally viewed as a disease of middle-aged and older adults, T2DM has seen the greatest relative increase in incidence and prevalence among young adults (under 40-years), adolescents, and children since the turn of the century.^2^ In 2019, the global age-standardized incidence rate of T2DM among adolescents and young adults was 183.36 per 100,000 population.^3^ Early onset of T2DM results in prolonged hyperglycemia and accelerated disease progression, significantly increasing the risk of cardiovascular disease and mortality compared to late-onset diabetes.^4^ Thus, timely identification of new risk factors is essential to curtail the progression of diabetes and reduce associated mortality rates.

Building upon this context, the Systemic Immune Inflammation Index (SII), proposed by Hu et al., is an innovative biomarker derived from the counts of platelets, neutrophils, and lymphocytes.^5^ This index captures the balance between immune response and inflammation, thus accurately indicating inflammation severity.^6^ Consequently, SII has become a vital biomarker for evaluating Cardiovascular Disease (CVD) and overall mortality risk.^7^^,^.^8^

Previous research has emphasized SII's prognostic significance across different populations, especially among the elderly and individuals with existing cardiovascular diseases.^9^ Evidence suggests that diabetes is associated with low-grade systemic inflammation, which contributes to obesity and insulin resistance. Notably, studies have revealed a nonlinear association between SII and CVD mortality in prediabetic and nondiabetic groups, while in diabetic patients, a linear relationship with cardiovascular mortality has been observed.^10^

Despite these findings, there is a notable gap in research concerning the effects of high SII levels in non-elderly diabetic patients, a demographic increasingly impacted by diabetes and its cardiovascular complications. To fill this gap, the authors undertook a study to explore the relationship between SII levels and all-cause and CVD mortality in non-elderly diabetic adults with diabetes, utilizing a nationally representative sample of diabetic adults in the United States.

This research sought to explore the relationship between increased SII levels and all-cause and cardiovascular mortality among non-elderly diabetic adults in the United States. By offering crucial insights into the prognostic significance of SII within this demographic, the authors intend to improve the current comprehension of risk assessment and management strategies for non-elderly individuals with diabetes.

## Materials and methods

### Data sources

The National Health and Nutrition Examination Survey (NHANES) is a comprehensive, cross-sectional survey conducted across the United States. It utilizes complex, multistage probability sampling to obtain a representative sample of the noninstitutionalized civilian population to evaluate nutritional status and potential health risk factors. The study protocol received ethical approval from the National Centre for Health Statistics (NCHS) (Protocol #98–12, Protocol #2005–06, Protocol #2011–17, Protocol #2018–01) and adhered to STROBE guidelines for cross-sectional research reporting. http://www.cdc.gov/nchs/nhanes/index.htm

### Study population

**Fig. 1:**
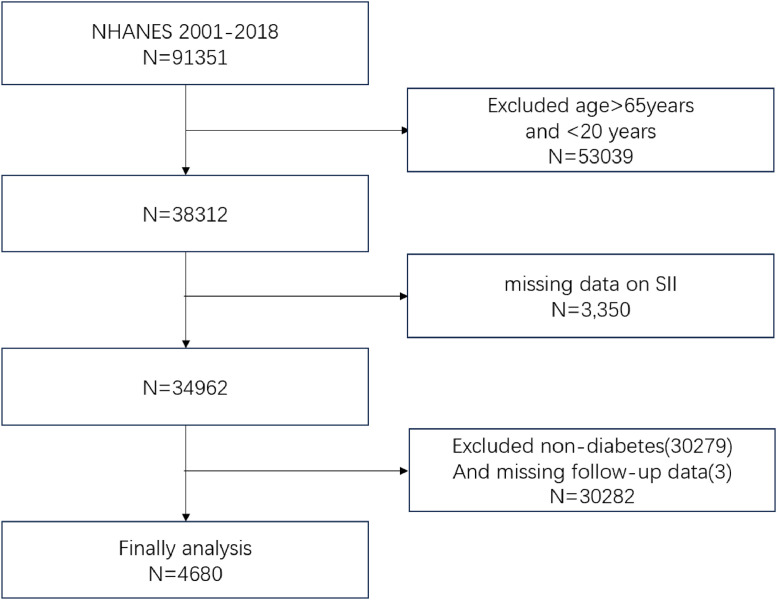
Flowchart for selecting participants from NHANES 2001–2008.

In this study, data from the NHANES database spanning 2001 to 2018 were analyzed. Among the 91,351 eligible individuals, exclusions were made for those aged 65-years or older, those under 20-years (*n* = 53,039), individuals lacking SII index data (*n* = 3350), those without diabetes (*n* = 1092), and those with missing follow-up data (*n* = 3). Consequently, the final analysis included 4680 participants. The sample selection process is depicted in Fig. 1.

### Definition of SII and diabetes

The study utilized the Systemic Immune-Inflammatory Index (SII) as its dependent variable. Lymphocyte, neutrophil, and platelet counts were determined using an automated hematology analysis device (CoulterDxH 800 analyzer), and SII was calculated using the formula platelet count × neutrophil count/lymphocyte count, as detailed in prior literature.^11^

Diabetes diagnosis criteria encompassed: 1) Fasting blood glucose ≥ 7.0 mmoL/L or an oral glucose tolerance test result ≥ 11.1 mmoL/L after two hours; 2) Random blood glucose ≥ 11.1 mmoL/L; 3) Glycated Hemoglobin (HbA1c) ≥ 6.5 %; 4) Insulin or diabetes medication usage; 5) Diagnosis by a healthcare professional.^12^

### Mortality assessment

Participants were tracked through the National Death Index up to December 31, 2019. Specific causes of death were determined using codes from the International Classification of Diseases, Tenth Revision (ICD-10). Mortality due to cardiovascular disease was identified by ICD-10 codes I00-I09, I11, I13, I20-I51, and I60-I69. The follow-up duration spanned from the baseline assessment to either death or the conclusion of the follow-up period.

### Covariates

The demographic variables considered were age, sex (male/female), race/ethnicity (non-Hispanic white, non-Hispanic black, Mexican American, other Hispanic, and other/multiracial), educational level (<9th grade, 9th‒11th grade, high school graduate, some college, and college graduate or higher), and poverty-to-income ratio (< 1.00, 1.00‒3.00, ≥ 3.00).

Lifestyle and health factors included waist circumference, Body Mass Index (BMI) category (normal < 25, overweight 25–30, and obese ≥ 30), smoking status (nonsmoker, former smoker, and current smoker), alcohol consumption (nondrinker, former drinker, light, moderate, and heavy drinker), and hypertension history (defined by: 1) Self-reported hypertension; 2) Use of antihypertensive medication; 3) Mean systolic blood pressure of at least 130 mmHg and/or mean diastolic blood pressure of at least 80 mmHg), diabetes medication use (categorized as insulin, oral antidiabetic drugs, or none), and antihypertensive medication use (yes or no).

Laboratory measures included glycosylated Hemoglobin (HbA1c), Serum creatinine (Scr), serum urea nitrogen (BUN), Uric Acid (UA), Low-Density Lipoprotein Cholesterol (LDL-C), Triglycerides (TG), Total Cholesterol (TC), and High-Density Lipoprotein Cholesterol (HDL-C).

### Statistical analysis

Survey analysis methods, incorporating sampling weights, were used to achieve nationally representative estimates. The baseline characteristics of the study population were summarized with means and Standard Deviations (SD) for continuous variables and frequencies and unweighted percentages for categorical variables. Analysis of Variance (ANOVA) assessed differences in continuous variables across multiple groups, while the Wilcoxon rank-sum test evaluated differences in ordinal variables between two independent groups. The Rao & Scott second-order corrected Chi-Square test was applied for categorical variables to ensure accurate analysis in complex sample studies.

The “maxstat” package (https://CRAN.R-project.org/package=maxstat) was used to determine the optimal SII cutoff point most significantly associated with survival outcomes by utilizing the maximum selected rank statistic. Participants were subsequently divided into high SII and low SII groups.

The association between SII and cardiovascular and all-cause mortality in non-elderly diabetic patients was evaluated using survey-weighted Cox regression analysis. Three models were constructed to account for potential confounders: Crude model was unadjusted; Model 1 was adjusted for sex, age, race, education level, and Poverty-to-Income Ratio (PIR); Model 2 further adjusted for BMI, alcohol consumption, smoking status, hypertension, Triglycerides (TG), Total Cholesterol (TC), High-Density Lipoprotein Cholesterol (HDL-C), Low-Density Lipoprotein Cholesterol (LDL-C), Serum creatinine (Scr), Blood Urea Nitrogen (BUN) levels, glycohemoglobin (HbA1c), use of antidiabetic drugs, and antihypertensive medications.

Restricted Cubic Splines (RCS) with three knots were employed to assess the nonlinear dose-response relationship between SII/SIRI and mortality outcomes, exploring potential nonlinear relationships between SII and both cardiovascular and all-cause mortality. Subgroup analyses based on age, sex, BMI, alcohol consumption, and smoking status were conducted to examine interactions between SII values and mortality. All statistical analyses were performed using *R* software version 4.3.2, with two-sided p-values <0.05 considered statistically significant.

## Results

### Baseline characteristics of participants

The study included 4680 diabetic patients, 52.62 % male, and an average age of 51.11 ± 0.21 years. During a median follow-up of 8.19-years, the all-cause mortality rate was 13.35 %, while the cardiovascular mortality rate was 3.46 %. Using the maximum selection rank statistic, the optimal SII threshold was determined to be 947.625, dividing participants into a lower SII group (≤ 947.625, *n* = 4212) and a higher SII group (> 947.625, *n* = 468). The higher SII group participants were more likely to be female and white. Detailed baseline characteristics are shown in Table 1A, Table 1B.

### Associations between SII and mortality

**Fig. 2:**
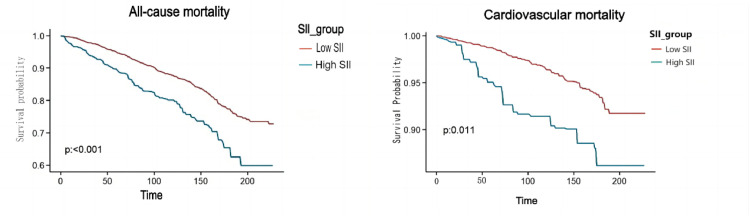
Weighted Kaplan-Meier plots illustrate SII's association with all-cause mortality and CVD mortality.

Table 2 illustrates that the crude model shows a significant link between SII and a higher risk of all-cause mortality (HR = 1.81, 95 % CI = 1.38‒2.37). After adjusting for various factors, each unit increase in SII correlated with a 103 % rise in all-cause mortality among diabetic patients (Model 1, HR = 2.04, 95 % CI = 1.54‒2.69) and a 99 % rise (Model 2, HR = 1.99, 95 % CI = 1.51‒2.62). Elevated SII was also notably linked to a higher risk of cardiovascular mortality: Crude model (HR = 2.42, 95 % CI = 1.47‒3.98, trend *p* < 0.001), Model 1 (HR = 2.90, 95 % CI = 1.82‒4.63, trend *p* < 0.001), and Model 2 (HR = 2.95, 95 % CI = 1.79‒4.86, trend *p* < 0.001). Kaplan-Meier analysis confirmed that the group with high SII had a reduced survival rate (Fig. 2).

**Table 2:** Hazard ratios of mortality according to the SII among non-elderly patients with diabetes.

Characteristic	Crude model		Model 1		Model 2	
	HR (95 % CI)	p	HR (95 % CI)	p	HR (95 % CI)	p
**All-cause mortality**						
SII	1.0004 (1.0002, 1.0007)	<0.001	1.0005 (1.0002, 1.0007)	<0.001	1.0004 (1.0002, 1.0006)	**<0.001**
**SII category**						
Lower SII (*n* = 4212)	Ref		Ref		Ref	
Higher SII (*n* = 468)	1.8101 (1.3806, 2.3731)	<0.001	2.0372 (1.5426, 2.6904)	<0.001	1.9931 (1.5145, 2.6230)	**<0.001**
**Cardiovascular mortality**						
SII	1.0007 (1.0004, 1.0010)	<0.001	1.0007 (1.0004, 1.0010)	<0.001	1.0006 (1.0004, 1.0009)	**<0.001**
**SII category**						
Lower SII (*n* = 4212)	Ref		Ref		Ref	
Higher SII (*n* = 468)	2.4156 (1.4674, 3.9763)	<0.001	2.9031 (1.8201, 4.6305)	<0.001	2.9500 (1.1.7912, 4,8584)	**<0.001**

Crude Model, Unadjusted; Model 1, Adjusted for age, sex, race, PIR, and educational level; Model 2, Adjusted for age, sex, race, PIR, educational level, smoking, drinking, hypertension, BMI, waist, HDL, LDL, TG, TC, UA, BUN, and Cr, Glycohemoglobin, Antidiabetic Drugs, Antihypertension Drugs.

### Dose-response relationship between SII and mortality

**Fig. 3:**
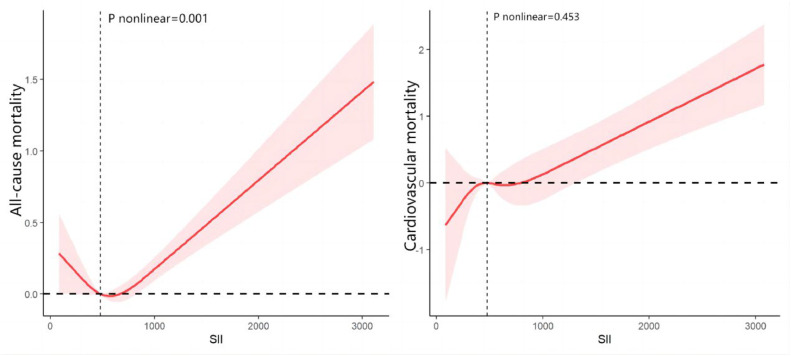
RCS analysis of the association between SII and mortality.

Fig. 3 depicts a nonlinear U-shaped relationship between SII and all-cause mortality after accounting for multiple potential confounders (*p* < 0.05). Conversely, no significant nonlinear relationship was found between SII and cardiovascular disease mortality (*p* = 0.068).

### Subgroup analysis

Subgroup analyses were performed based on gender, BMI, hypertension, and smoking status. The results showed that when the SII index surpasses the threshold, it is significantly linked to an elevated risk of all-cause mortality in women (HR = 2.05, 95 % CI 1.37–3.06), men (HR = 1.94, 95 % CI 1.34–2.79), obese individuals (HR = 1.80, 95 % CI 1.24–2.61), overweight individuals (HR = 2.68, 95 % CI 1.61–4.44), hypertensive patients (HR = 2.02, 95 % CI 1.45–2.81), current smokers (HR = 2.03, 95 % CI 1.23–3.34), and former smokers (HR = 2.34, 95 % CI 1.33–4.12). No significant interactions were found between SII and the stratification variables mentioned (p-interaction > 0.05) (Table 3).

**Table 3:** Subgroup analysis of the associations between SII and mortality among the young population with diabetes.

Subgroup	All-cause mortality			Cardiovascular mortality		
	HR (95 % CI)	p	p interaction	HR (95 % CI)	p	p interaction
**Sex**			0.942			0.269
**Male**	1.93 (1.34, 2.79)	**<0.001**		2.44 (1.25, 4.79)	**0.009**	
**Female**	2.05 (1.37, 3.06)	**<0.001**		4.71 (2.22, 9.99)	**<0.001**	
**Smoking**			0.576			0.625
Never	1.79 (1.14, 2.82)	**0.011**		2.68 (1.22, 5.86)	**0.014**	
Former	2.34 (1.33,4.12)	**0.003**		5.34 (1.96, 14.58)	**0.001**	
Now	2.03 (1.23, 3.34)	**0.005**		3.14 (1.37, 7.19)	**0.006**	
**Hypertension**			0.724			0.884
No	1.85 (0.918,3.79)	0.091		2.82 (0.70, 11.29)	0.144	
Yes	2.02 (1.45, 2.81)	**<0.001**		2.90 (1.68, 5.02)	**<0.001**	
**BMI**			0.563			0.866
Normal	2.01 (0.85, 4.77)	0.111		2.97 (0.66, 13.35)	0.155	
Overweight	2.68 (1.61, 4.44)	**<0.001**		5.02 (2.09, 12.05)	**<0.001**	
Obesity	1.80 (1.24, 2.61)	**0.002**		3.06 (1.64, 5.71)	**<0.001**	

SII, Systemic Immune Inflammatory Index; BMI, Body Mass Index; HR, Hazard Ratio; CI, Confidence Interval.

Similarly, regarding cardiovascular disease mortality, when the SII index was above the threshold, significant associations were observed in women (HR = 4.71, 95 % CI 2.22–9.99), men (HR = 2.44, 95 % CI 1.25–4.79), obese individuals (HR = 3.06, 95 % CI 1.64–5.71), overweight individuals (HR = 5.02, 95 % CI 2.10–12.05), hypertensive patients (HR = 2.90, 95 % CI 1.68–5.02), current smokers (HR = 3.14, 95 % CI 1.37–7.19), and former smokers (HR = 5.34, 95 % CI 1.96–14.58). Interaction tests revealed that the stratification variables did not significantly affect this positive association (all p-values for interactions > 0.05).

## Discussion

With global economic advancement, there is an increasing prevalence of obesity, high consumption of unhealthy foods, and lack of physical activity, leading to a rise in early-onset type 2 diabetes.^13^ This trend has significantly exacerbated the disease burden. The earlier the onset of diabetes, the longer the duration of hyperglycemia, and the more rapidly pathological processes, such as severe insulin resistance and β-cell dysfunction progress.^14^ This results in poorer glycemic control and an elevated risk of diabetic complications. Patients with early-onset diabetes face a higher risk of complications, markedly increasing their susceptibility to cardiovascular disease and mortality.^15^ Understanding the fundamental causes of early-onset type 2 diabetes is essential for mitigating complications and enhancing the quality of life for patients, thus reducing the socioeconomic burden.

Previous research has shown that elevated SII levels increase the risk of developing diabetes and negatively impact the prognosis of diabetic patients.^16^^,^^17^ However, there is a paucity of studies assessing the SII score and its prognostic implications in non-elderly diabetic populations. This study examines the relationship between SII and all-cause mortality, as well as Cardiovascular Disease (CVD) mortality, in non-elderly diabetic individuals. These results demonstrate that higher SII levels are significantly associated with an increased risk of all-cause and CVD mortality, irrespective of demographic, socioeconomic, and lifestyle factors.

The SII is calculated from peripheral blood counts of Platelets (P; × 10^9/L), Neutrophils (N; × 10^9/L), and Lymphocytes (L; × 10^9/L) using the formula: SII = *P* × *N*/L.^18^ This index acts as a comprehensive measure of the balance between pro-inflammatory and anti-inflammatory cellular responses.^19^ It encompasses pathways involved in thrombosis, inflammation, and adaptive immunity.^20^ Elevated SII levels indicate heightened systemic inflammation, which can detrimentally affect various physiological functions and accelerate disease progression.

SII has gained recognition as a potential prognostic marker for several clinical conditions. In oncology, elevated SII levels correlate with poorer prognoses in multiple cancer types, including solid tumors such as breast^21^, pancreatic,^22^ lung,^23^ and cervical cancers,^24^ reflecting an imbalance in systemic inflammation and immune responses. In cardiovascular disease, high SII is associated with an increased risk of adverse cardiac events, highlighting the role of inflammation in the pathogenesis and progression of atherosclerosis.^25^, ^26^, ^27^, ^28^ Furthermore, SII has shown predictive value for mortality in diabetic patients, indicating its utility in identifying individuals at higher risk for complications and death.^9^^,^^29^ These findings highlight the potential of SII as a valuable marker across various diseases, especially where inflammation is a key factor.

Inflammation is integral to the pathophysiology of diabetes and its associated complications.^30^ Chronic inflammation triggers intracellular inflammatory signaling pathways, worsening insulin resistance and beta-cell dysfunction^31^, which are pivotal in developing and progressing type 2 diabetes. Furthermore, systemic inflammation is correlated with increased endothelial dysfunction, atherosclerosis, and thrombosis^32^, which elevate mortality rates, particularly due to cardiovascular events. Chronic inflammation is also a critical factor in the pathogenesis of cardiovascular diseases. Research has shown that anti-inflammatory therapies for diabetes can enhance glycemic control and improve insulin secretion.^33^

Inflammation plays a crucial role in the pathophysiology of diabetes and obesity, which is a major driver and is particularly prevalent in non-elderly patients. A higher proportion of these patients are affected by obesity and a lack of physical activity.^15^ In obese individuals, the expansion of adipose tissue results in increased serum fatty acid levels and decreased oxygen content in adipocytes.^34^ This induces chronic inflammation in adipose tissue through direct or indirect pathways, exacerbating insulin resistance. Studies indicate that immune cell accumulation and inflammatory cytokines are characterized by pancreatic inflammation, predominantly driven by pro-inflammatory M1 macrophages.^35^

In addition, the present study found that a higher proportion of women were present in the elevated SII group (Table 1). This observation may suggest gender-based differences in immune-inflammatory responses, which could be influenced by sex hormones such as estrogen or progesterone. These hormonal differences, particularly across the menopausal transition, have been shown to modulate inflammatory activity and vascular risk. However, due to substantial missing data (41 %) in the menopausal status variable, the authors could not conduct subgroup analysis or adjust the models based on menopausal status. The authors have acknowledged this limitation explicitly and encourage future research to examine this important aspect with more complete reproductive health information.

Research demonstrates that myeloid cell numbers, particularly monocytes and macrophages, significantly increase in the islets of obese animal models and type 2 diabetes patients. Ehses et al. observed a substantial increase in macrophage numbers in the pancreases of HFD-fed mice and GK rats, accompanied by a significant release of inflammatory cytokines such as IL-6, IL-8, CXCL1, G-CSF, and MIP1a.^36^ Similarly, Cucak et al., in their study of db/db mice, found notable macrophage accumulation in the pancreas of diabetic mice, which displayed a pro-inflammatory M1-like phenotype.^37^ Eguchi et al. reported that saturated fatty acids induce β-cells to produce chemokines, attracting pro-inflammatory monocytes/macrophages to the islets.^38^ Ying et al. indicated that obesity-related pancreatic inflammation is primarily mediated by macrophages, with a lesser role for adaptive immune cells.^35^ These findings underscore the critical roles of macrophages and inflammatory cytokines in developing obesity and diabetes. Consequently, the characteristics often associated with this cohort of non-elderly diabetic patients, such as higher rates of obesity, contribute to a pronounced immune response marked by elevated inflammatory markers. This heightened inflammatory state can promote insulin resistance and β-cell dysfunction, potentially accelerating the progression of diabetic complications.

In this study, the authors investigated the prognostic value of SII in non-elderly patients with diabetes. The present results showed that elevated SII levels were significantly associated with increased risks of all-cause and Cardiovascular Disease (CVD) mortality in this population. Specifically, higher SII was associated with a 0.99-fold increased risk of all-cause mortality and a 1.95-fold increased risk of CVD mortality, independent of demographic, socioeconomic, and lifestyle factors. In addition, RCS curve analysis revealed a U-shaped nonlinear relationship between SII and all-cause mortality, indicating that very low and very high SII were associated with a higher mortality risk. In contrast, the relationship between SII and CVD mortality was linear, indicating a continuous increase in risk with increasing SII levels. In light of these findings, SII is expected to be a valuable indicator for predicting mortality risk in non-elderly patients with diabetes. It is able to identify individuals at higher risk of all-cause and CVD mortality, thereby facilitating early risk stratification and targeted interventions, which could potentially slow the progression of diabetes and its complications, ultimately improving patient outcomes. Early identification of high-risk individuals through SII may allow healthcare providers to implement more aggressive surveillance and preventive measures, potentially slowing the progression of diabetes and its complications.

### Limitations

Several limitations must be acknowledged when interpreting the present findings. First, the study's observational design establishes a causal relationship between the SII and mortality outcomes. Second, although the authors adjusted for various known confounders in these analyses, residual confounders cannot be completely excluded. Confounding from unmeasured or unknown variables may still influence the observed associations. Third, SII was measured only once at baseline, which fails to capture potential temporal fluctuations in inflammatory status that may be critical for long-term prognosis. Fourth, as the study population primarily comprised non-elderly diabetic adults, the generalizability of these results to older individuals or to those without diabetes, who may display markedly different metabolic and inflammatory profiles, remains limited. Finally, the authors’ attempt to assess the impact of menopausal status in female participants was hampered by substantial missing data (41 %), thereby constraining the ability to elucidate sex-specific differences in the observed associations fully.

## Conclusions

In conclusion, this study highlights a significant association between SII and increased all-cause and cardiovascular mortality in non-elderly patients with diabetes. These findings highlight the potential of SII as a prognostic marker and the importance of addressing systemic inflammation to improve mortality outcomes in this population. Continued research in this area is essential to develop effective strategies to reduce the risk of inflammation-related mortality. Table 1a and 1b

**Table 1A:** Baseline characteristics of the study population (categorical variables).

Characteristic	Total (*n* = 4680)	Lower SII (*n* = 4212)	Higher SII (*n* = 468)	p-value
**Sex**, n ( %)				**0.001**
Male	2384 (52.62)	2184 (53.87)	200 (42.96)	
Female	2296 (47.38)	2028 (46.13)	268 (57.04)	
**Race**, n ( %)				**<0.001**
Mexican American	1052 (11.41)	963 (11.79)	89 (8.51)	
Non-Hispanic Black	1266 (15.93)	1164 (16.46)	102 (11.85)	
Non-Hispanic White	1338 (56.56)	1155 (55.29)	183 (66.33)	
Other Hispanic	500 (6.60)	451 (6.59)	49 (6.60)	
Other Race	524 (9.51)	479 (9.87)	45 (6.72)	
**Education levels**, n ( %)				0.56
<9th grade	742 (8.63)	686 (8.93)	56 (6.35)	
9‒11th grade	777 (13.21)	692 (13.11)	85 (13.97)	
High school graduate/GED or equivalent	1080 (25.09)	963 (24.98)	117 (25.96)	
Some college or AA degree	1329 (32.16)	1187 (31.88)	142 (34.29)	
College graduate or above	752 (20.91)	684 (21.10)	68 (19.44)	
**BMI category**, n ( %)				0.16
Normal (< 24.9)	535 (10.38)	464 (9.91)	56 (11.09)	
Overweight (25–30)	1226 (23.24)	1119 (23.50)	107 (21.25)	
Obesity (≥ 30)	2919 (66.38)	2619 (66.34)	300 (66.68)	
**PIR category**, n ( %)				0.16
≤ 1.0	1201 (18.71)	1067 (18.50)	134 (20.35)	
1.0–3.0	1960 (36.62)	1776 (37.26)	184 (31.68)	
> 3.0	1519 (44.67)	1369 (44.24)	150 (47.97)	
**Smoking**, n ( %)				0.54
Former	1244 (27.91)	1123 (28.20)	121 (25.67)	
Never	2408 (50.19)	2170 (50.22)	238 (49.96)	
Now	1028 (21.90)	919 (21.58)	109 (24.38)	
**Drinking**, n ( %)				0.91
Former	1044 (20.34)	935 (20.47)	109 (19.39)	
Heavy	829 (17.51)	748 (17.30)	81 (19.13)	
Mild	1424 (33.56)	1295 (33.55)	129 (33.65)	
Moderate	596 (14.55)	529 (14.50)	67 (14.98)	
Never	787 (14.04)	705 (14.19)	82 (12.85)	
**Hypertension**, n ( %)				0.2
No	1649 (36.88)	1501 (37.37)	148 (33.15)	
Yes	3031 (63.12)	2711 (62.63)	320 (66.85)	
**Antidiabetic Drugs**: n ( %)				0.12
Insulin	878 (18.69)	761 (18.04)	117 (23.64)	
Oral	1949 (41.59)	1759 (42.06)	190 (38.00)	
None	1853 (39.72)	1692 (39.90)	161 (38.35)	
**Antihypertension Drugs**: n ( %)				0.38
Yes	2596 (55.47)	2322 (55.14)	274 (58.24)	
No	2081 (44.48)	1887 (44.86)	194 (41.76)	

BMI, Body Mass Index; PIR, Family Income-poverty Ratio.

**Table 1B:** Baseline characteristics of the study population (continuous variables).

Characteristic	Total (*n* = 4680)	Lower SII (*n* = 4212)	Higher SII (*n* = 468)	p-value
**Age**, years	51.11 ± 0.21	51.21 ± 0.22	50.34 ± 0.73	0.26
**BMI**, kg/m^2^	34.01 ± 0.19	33.89 ± 0.19	34.91 ± 0.62	0.11
**Waist**, cm	112.23 ± 0.42	111.95 ± 0.46	114.35 ± 1.28	0.09
**Creatinine**, mg/dL	0.91±0.01	0.90 ± 0.01	0.98 ± 0.04	0.06
**HbA1c**, %	7.28 ± 0.04	7.30 ± 0.04	7.11 ± 0.12	0.14
**Blood Urea Nitrogen**, mg/dL	14.21 ± 0.12	14.16 ± 0.13	14.61 ± 0.45	0.35
**Uric acid**, mg/dL	5.61 ± 0.03	5.61 ± 0.03	5.59 ± 0.12	0.90
**Triglycerides**, mg/dL	209 ± 4.4	211 ± 4.7	193 ± 10.1	0.10
**Total cholesterol**, mg/dL	193 ± 1.0	194 ± 1.0	186 ± 3.1	**0.02**
**HDL-C**, mg/dL	46 ± 0.3	46 ± 0.3	48 ± 1.0	0.05
**LDL-C**, mg/dL	107 ± 0.7	108 ± 0.7	101 ± 2.7	**0.03**
**PIR**	2.80±0.04	2.79±0.04	2.89±0.12	0.44

BMI, Body Mass Index; HbA1c, Glycosylated Hemoglobin A1c; HDL-C, High-Density Lipoprotein Cholesterol; LDL-C, Low-Density Lipoprotein Cholesterol; PIR, Family-Income-Poverty Ratio.

## Data availability

The data supporting this study's findings are available from the NHANES database, which can be accessed at https://www.cdc.gov/nchs/nhanes/index.htm.

## Authors’ contributions

B.H: Conceptualization, Writing - original draft. T.L: Formal analysis, Writing - review & editing. Y.S: Software, Formal analysis, Data curation. J.S: Investigation, Writing - review & editing. L.F: Supervision, Methodology, Resources. F.L: Supervision, Project administration.

## CRediT authorship contribution statement

**Bing Hu:** Conceptualization, Writing – original draft. **Tong Liu:** Formal analysis, Writing – review & editing. **Yanxiang Sun:** Software, Formal analysis, Data curation. **Jie Sun:** Investigation, Writing – review & editing. **Li Feng:** Supervision, Methodology, Resources. **Fei Li:** Supervision, Project administration.

## Declaration of competing interest

The authors declare no conflicts of interest.
